# A framework of biomarkers for adipose tissue aging: a consensus statement by the Aging Biomarker Consortium

**DOI:** 10.1093/lifemedi/lnaf027

**Published:** 2025-07-19

**Authors:** Aging Biomarker Consortium, Jian Yu, Yankang Zhang, Ting Zhang, Yan Bi, Yan Chen, Zheng Chen, Zhe Dai, Feifan Guo, Lixin Guo, Cheng Hu, Xiangqing Kong, Jian Li, Pingsheng Liu, Yong Liu, Jing Qu, Qiqun Tang, Congyi Wang, Liheng Wang, Jiqiu Wang, Jianping Weng, Aimin Xu, Lingyan Xu, Huijie Zhang, Jiajun Zhao, Jingjing Zhang, Weiqi Zhang, Tongjin Zhao, Weiping Zhang, Zhiming Zhu, Guang-Hui Liu, Guang Ning, Gang Pei, Li Qiang, Feng Liu, Xinran Ma

**Affiliations:** School of Life Sciences, East China Normal University, Shanghai 200241, China; School of Life Sciences, East China Normal University, Shanghai 200241, China; School of Life Sciences, East China Normal University, Shanghai 200241, China; Nanjing Drum Tower Hospital, Medical School of Nanjing University, Nanjing 210008, China; Shanghai Institute of Nutrition and Health, Chinese Academy of Sciences, Shanghai 200031, China; School of Life Science and Technology, Harbin Institute of Technology, Harbin 150001, China; Endocrinology Department of Zhongnan Hospital, Wuhan University, Wuhan 430071, China; Institute for Translational Brain Research, Fudan University, Shanghai 200032, China; Institute of Geriatric Medicine, Chinese Academy of Medical Sciences, Beijing 100730, China; Shanghai Sixth People’s Hospital Affiliated to Shanghai Jiao Tong University School of Medicine, Shanghai 200233, China; The First Affiliated Hospital of Nanjing Medical University, Nanjing 210029, China; Institute of Geriatric Medicine, Beijing Hospital, Beijing 100730, China; Institute of Biophysics, Chinese Academy of Sciences, Beijing 100101, China; College of Life Sciences, Wuhan University, Wuhan 430072, China; Beijing Institute of Stem Cell and Regenerative Medicine, Beijing 100101, China; School of Basic Medical Sciences, Fudan University, Shanghai 200032, China; Tongji Hospital, Tongji Medical College, Huazhong University of Science and Technology, Wuhan 430030, China; Institute of Cardiovascular Sciences, Peking University, Beijing 100191, China; Ruijin Hospital Affiliated to Shanghai Jiao Tong University School of Medicine, Shanghai 200025, China; Anhui Medical University, Hefei 230032, China; School of Clinical Medicine, The University of Hong Kong, Hong Kong 999077, China; School of Life Sciences, East China Normal University, Shanghai 200241, China; Nanfang Hospital, Southern Medical University, Guangzhou 510515, China; Shandong Provincial Hospital Affiliated to Shandong First Medical University, Jinan 250021, China; The Second Xiangya Hospital of Central South University, Changsha 410011, China; Beijing Institute of Genomics, Chinese Academy of Sciences, Beijing 100101, China; Academy of Medical Sciences, Zhengzhou University, Zhengzhou 450001, China; School of Basic Medical Sciences, Naval Medical University, Shanghai 200433, China; Daping Hospital, Army Medical University, Chongqing 400042, China; Institute of Zoology, Chinese Academy of Sciences, Beijing 100101, China; Ruijin Hospital Affiliated to Shanghai Jiao Tong University School of Medicine, Shanghai 200025, China; College of Life Sciences and Technology, Tongji University, Shanghai 200092, China; School of Basic Medical Sciences, Peking University, Beijing 100191, China; The Second Xiangya Hospital of Central South University, Changsha 410011, China; School of Life Sciences, East China Normal University, Shanghai 200241, China; Institute for Aging, East China Normal University, Shanghai 200062, China

## Abstract

Adipose tissue serves as a crucial energy storage and metabolic organ in the human body. With the surging of elderly population in China comes significant challenges in preventing and managing age-associated diseases, while adipose tissue aging represents one of the pivotal initiating events for multi-organ senescence. To address these challenges, the Aging China Biomarkers Consortium (ABC) has established an expert consensus on biomarkers of adipose tissue aging by digesting literature and collecting insights from scientists and clinicians. This consensus provides a comprehensive evaluation of the key changes and characteristics, as well as biomarkers related to adipose tissue aging and proposes a systematic framework categorizing these biomarkers into functional, structural and humoral dimensions. Within each dimension, the ABC recommends clinically and empirically validated biomarkers and parameters for assessing both physiological and pathological changes in adipose tissue during aging, which aims to establish a foundation for future prediction, diagnosis, early warning and treatment for adipose tissue aging and its related diseases, with the ultimate goal of improving adipose tissue health and promoting healthy aging in elderly populations both in China and worldwide.

## Introduction

Adipose tissue is one of the most critical organs for energy homeostasis in mammals. It features high plasticity and regulates systematic metabolic balance through energy storage (primarily as triglycerides) and expenditure in responding to external stimuli such as temperature and nutrients. Adipose tissue can be classified into various types based on distinct anatomical location, morphology, and function. Adult humans possess diverse white adipose depots, including subcutaneous, intra-abdominal visceral, perirenal, pericardial, and mesenteric fat, as well as thermogenic brown and beige fat in the neck, supraclavicular, scapular, and spinal regions. Additionally, specialized fat depots exist, such as pink adipose tissue in lactating mammary glands and yellow fat in bone marrow. These distinct adipose tissues undergo phenotypic and functional transformations under different physiological and pathological conditions. Furthermore, as an endocrine organ, the ability of adipose tissues secreting adipokines (e.g. leptin, adiponectin) and cytokines fluctuates in aging, thus impacting systemic energy balance, food intake, lipid and glucose homeostasis, as well as thermogenesis and immune responses [1].

With the advance of age, adipose tissue undergoes natural aging. Meanwhile, obesity accelerates adipose tissue aging. Although the molecular driving force for adipose tissue aging during natural aging and obesity differs, they share similar pathological features, including mitochondrial dysfunction, endoplasmic reticulum stress, dysregulated nutrient sensing, and increased production/secretion of pro-inflammatory/pro-fibrotic factors. These changes ultimately lead to reduced tissue plasticity, functional decline, and systemic metabolic imbalance. Adipose tissue is highly heterogeneous, comprising multiple cell types, such as mature adipocytes, preadipocytes, progenitor cells, vascular endothelial cells, and various immune cells. During aging, functional alterations occur across various cell populations. For example, mature adipocytes exhibit diminished lipid storage and thermogenic capacity; preadipocytes and progenitor cells show reduced proliferative and differentiation potential; vascular endothelial cell proliferation is impaired, leading to tissue hypoxia; and immune cell infiltration is heightened, exacerbating chronic low-grade inflammation. These cellular changes interact with and impact each other, collectively driving tissue aging.

Recent studies highlight that adipose tissue aging is one of the pivotal initiating events in multi-organ aging, promoting the senescence of other organs through inter-organ communications, thereby compromising overall health. For instance, adipose tissue aging is a significant risk factor for cardiovascular diseases, metabolic syndrome, and neurodegenerative disorders. Moreover, adipose tissue serves as the key therapeutic target for multiple anti-aging interventions. Identification of biomarkers for adipose tissue aging will deepen our understanding of the hallmarks of this critical event, thereby enabling early prediction/diagnosis, guiding personalized therapeutic strategies, and facilitating dynamic monitoring of treatment efficacy, which ultimately mitigates the onset and progression of multiple aging-related diseases by targeting adipose tissue aging. Thus, defining adipose aging biomarkers holds substantial research and clinical value.

On 1 March 2025, the Aging Biomarker Consortium (ABC) of China gathered experts of adipose tissue research and aging studies for a workshop hosted by the Institute of Aging Research at East China Normal University, Shanghai, China [2, 3]. Based on published literature, peer-reviewed research foundations, evidence-based medical data, and expert opinions, the consortium established an expert consensus recommending biomarkers that reflect adipose tissue aging. These biomarkers are intended to assess individual adipose tissue aging status and rate, predict risks of adipose aging-related diseases, and provide a scientific evaluation system for delaying and intervening adipose tissue aging and its associated diseases, ultimately aiming to extend lifespan and healthspan.

## Methodology for recommended biomarkers of adipose tissue aging

We conducted a comprehensive literature search on well-established databases, including MEDLINE, PubMed, and Cochrane Library, along with other relevant sources closely related to this consensus. For detailed descriptions of the specific search terms used, readers are encouraged to consult the online data supplement, which contains the final evidence tables summarizing the information used by the consensus writing group to formulate recommendations. Based on the integration of existing publications and collective research, ABC members collaboratively identified key questions regarding adipose tissue aging biomarkers through online collaboration. Subsequently, the identified biomarkers underwent thorough deliberation during face-to-face meetings to reach a unified consensus. ABC members meticulously reviewed and discussed all recommendations, thereby encompassing the multidimensional perspectives and considerations articulated in this consensus document. As shown in Table 1, we adhered to internationally recognized conventions for expressing evidence levels and recommendation strengths.

**Table 1.** Classification and definition of levels of evidence and levels of recommendation

Class (strength) of recommendation	Level (quality) of evidence
**Class I (Strong) Benefit >>> Risk** Suggested phrases for writing recommendations: • Is recommended • Is indicated/useful/effective/beneficial • Should be performed/administered/other	**Level A** Data derived from multiple randomized clinical trials or meta-analyses
**Class Ila (Moderate) Benefit >> Risk** Suggested phrases for writing recommendations: • Is reasonable • Can be useful/effective/beneficial	**Level B** Data derived from a single randomized clinical trial or large non randomized studies
**Class IIb (Weak) Benefit > Risk** Suggested phrases for writing recommendations: • May/might be reasonable • May/might be considered • Usefulness/effectiveness is unknown/unclear/uncertain or not well established	**Level C** Consensus of expert opinion, and/or small studies, retrospective studies, registries
**Class Ill: Harm (Strong) Risk > Benefit** Suggested phrases for writing recommendations: • Potentially harmful • Causes harm • Associated with excess morbidity/mortality • Should not be performed/administered/other	**Note:** COR and LOE are determined independently (any COR may be paired with any LOE). COR, class of recommendation; LOE, level of evidence.

## Classification and clinical application of adipose tissue aging biomarkers

**Figure 1.**
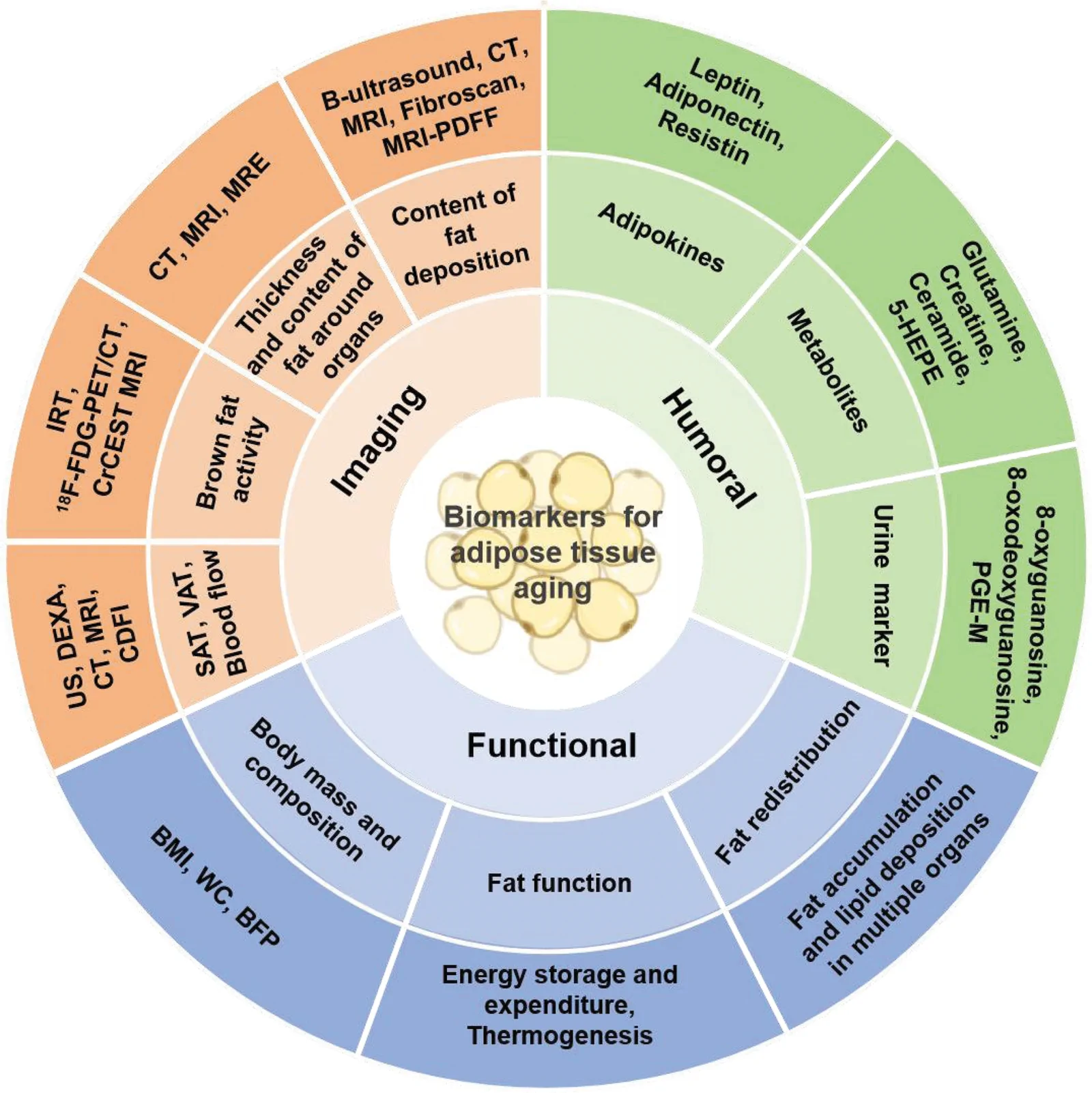
Biomarker framework for adipose tissue aging biomarkers. The proposed framework for assessing adipose tissue aging comprises three dimensions: functional biomarkers, imaging biomarkers, and body fluid biomarkers. This framework encompasses the diverse changes that occur across all levels of the adipose tissue during aging. These biomarkers show promising potential for widespread application in routine clinical practice. However, it should be emphasized that additional validation studies are required to evaluate the efficacy of these biomarkers in assessing adipose tissue aging. The abbreviations used in Fig. 1 correspond to those defined in Table 3. The figure is created with BioRender.com.

Adipose tissue aging involves multidimensional alterations at the molecular, cellular, organ, individual, and population levels. Importantly, given the diversity in adipose tissue distribution, subtypes, and functions within the body, as well as its significant role in systemic aging as well as the aging of adjacent organs, a detailed classification is necessary. Adipose aging biomarkers refer to indicators that accurately reflect “true adipose age,” “adipose structure,” and “adipose function.” These biomarkers can be used to assess the degree and rate of adipose aging, evaluate disease risk, and monitor the efficacy of anti-aging interventions. This consensus selects biomarkers from three dimensions—functional, imaging-based, and fluid-based for different adipose depots, serving as reference for clinical practice and future research (Fig. 1).

## Functional markers

### White adipose tissue (WAT) accumulation at early-stage of aging

In the early stages of aging, both visceral (VAT) and subcutaneous adipose tissue (SAT) exhibit accumulation. VAT accumulation is the most common manifestation during middle age, typically accompanied by increased abdominal circumference and is more prevalent in males (apple-shaped obesity). The primary change in adipose depots during early middle and old age is VAT expansion. VAT expansion serves as an independent risk marker for metabolic diseases (e.g. type 2 diabetes, fatty liver), cardiovascular disorders (e.g. atherosclerosis), cancer, and mortality [4–6]. Higher visceral fat content is also associated with thinner cerebral cortices and poorer cognitive function [7, 8]. Concurrently, excessive subcutaneous fat accumulation, particularly in the hips, thighs, and upper arms (pear-shaped obesity, more common in females), is associated with a relatively lower metabolic risk.

Body mass index (BMI) is a commonly used metric for assessing obesity. Abnormal BMI (high or low) may indicate dysregulated fat storage or metabolic function. In China, a BMI of 24.0–27.9 kg/m² is classified as overweight, ≥ 28.0 kg/m² as obese, and < 18.5 as underweight or indicative of malnutrition [9]. Studies show that in the elderly, a high BMI (≥ 30 kg/m²) correlates with an increased risk of aging-related diseases and accelerated aging markers [10]. However, BMI alone does not fully reflect the severity of obesity or adipose tissue dysfunction.

Body fat percentage (BFP) directly reflects the proportion of adipose tissue to total body weight and is a critical clinical indicator for assessing adipose dysfunction. Common BFP measurement methods include: skinfold thickness (simple but limited accuracy), bioelectrical impedance analysis (BIA; convenient but influenced by hydration status), dual-energy X-ray absorptiometry (DEXA; gold standard for clinical use), computed tomography (CT), and magnetic resonance imaging (MRI). A BFP exceeding 25% (males) or 30% (females) is generally considered excessive. However, BFP ranges vary widely and naturally increase with age, particularly in females. Elevated BFP is associated with higher mortality and frailty in the elderly [11–13].

Recent studies have revealed that metabolic resilience, defined as the organism’s ability to respond to energy imbalance and restore baseline metabolic homeostasis, shows a significant decline in both aging and obesity. To systematically evaluate this capacity, researchers developed the Gene Elasticity Score (GElaS) to quantify physiological and transcriptomic resilience in metabolically active tissues of mice and non-human primates. PPARγ was identified as the primary transcriptional regulator of elasticity-related genes in adipose tissue. Further studies demonstrate that modulating PPARγ activity enhances metabolic flexibility, improves metabolic health, and extends lifespan in mice [14, 15]. This provides a novel potential strategy for delaying aging and counteracting obesity-related metabolic decline.

### Changes in distribution abdominal and subcutaneous adipose tissue and adipose tissue atrophy in late-stage aging

In advanced aging, adipose tissue undergoes significant redistribution. In younger individuals, the total volume of SAT typically exceeds that of VAT, but subcutaneous fat gradually decreases with age. This reduction is primarily attributed to increased mature adipocyte death and depletion of adipocyte progenitor cell pools, which may be closely associated with aging mechanisms, such as diminished progenitor cell proliferation capacity and telomere shortening [16]. The loss of subcutaneous fat and impaired lipid storage capacity lead to ectopic lipid deposition in abdominal visceral depots, muscles, liver, bone marrow, skeletal muscle, heart, and pancreatic islets. This ectopic lipid distribution not only disrupts the metabolic buffering function of adipose tissue but may also cause localized tissue dysfunction, thereby increasing the risk of metabolic syndromes such as insulin resistance, hypertriglyceridemia, hypercholesterolemia, and cardiovascular diseases in elderly individuals. In some individuals with lipid metabolism disorders, the body gradually loses the ability to form sufficient subcutaneous fat for lipid storage, particularly in the lower body—a phenomenon termed subcutaneous lipodystrophy or adipose atrophy. This condition leads to severe adipose tissue inflammation and fibrosis and is closely associated with the development of severe metabolic diseases and increased mortality [17].

Both BMI and overall body fat percentage have limitations in accurately reflecting body fat distribution and aging status [5]. Waist circumference or waist-to-hip ratio can indicate central obesity. Waist circumference is a commonly used indicator of central obesity, defined in China as ≥ 90 cm for males or ≥ 85 cm for females. Waist-to-hip ratio is another indicator of central obesity, with diagnostic thresholds of ≥ 0.90 for males or ≥ 0.85 for females. With aging, both waist circumference and waist-to-hip ratio show gradual increases. Research demonstrates that BMI, waist circumference, and waist-to-hip ratio are significantly associated with all-cause and cardiovascular mortality in both genders, with waist circumference and waist-to-hip ratio reflecting higher risks of all-cause and cardiovascular mortality [18]. The visceral adiposity index, constructed using waist circumference, BMI, triglycerides, and high-density lipoprotein (HDL)-cholesterol, can predict cardiovascular and metabolic health risks [19, 20].

Based on the relative proportions of abdominal and subcutaneous adipose tissue, researchers have proposed the concepts of metabolically healthy obesity (MHO) and metabolically unhealthy obesity (MUO). Compared to MUO, MHO exhibits less ectopic fat deposition, increased lower-body fat distribution, better SAT expandability, insulin sensitivity comparable to non-obese healthy individuals, superior β-cell function, and better cardiorespiratory fitness. Studies have identified several key distinguishing factors between MHO and MUO: differences in body fat distribution (reduced intra-abdominal fat with increased lower-body fat), plasma adiponectin and PAI-1 concentrations, oxidative stress levels, β-cell function, skeletal muscle biology (reduced ceramide content with increased expression of genes involved in mitochondrial structure and function), 24-hour plasma substrate (glucose, NEFA, and triglyceride) and insulin concentrations, and adipose tissue biology (reduced expression of genes involved in inflammation and ECM remodeling with increased expression of adipogenic genes). These factors help evaluate the cardiometabolic health status of obese patients [21].

Recent studies have identified an “obesity paradox” where obesity may serve as a protective factor for health and survival in specific populations (e.g. very elderly individuals, or those with wasting diseases or infectious diseases). For instance, a study of individuals aged 80 years or older found that the apparent inverse association between higher waist circumference and reduced mortality risk was spurious—in reality, increasing waist circumference was associated with elevated risks of all-cause, cardiovascular, and non-cardiovascular mortality. Furthermore, combined analysis of BMI and waist circumference revealed that individuals with high BMI but low waist circumference had the lowest mortality risk. These findings emphasize that BMI may not be the optimal indicator for assessing obesity, while waist circumference may be more meaningful for health evaluation, particularly in elderly populations.

### Pathological changes in visceral adipose tissue

Adipocytes in VAT are characterized by large unilocular lipid droplets and relatively few mitochondria. These cells respond to systemic energy fluctuations through lipid storage and release, with particularly strong catecholamine-regulated lipolytic capacity. During aging, VAT typically exhibits adipocyte hypertrophy and increased tissue inflammation, leading to systemic insulin resistance, dyslipidemia, and overall metabolic dysregulation [5]. Starting at middle age, adults often suffer from visceral adiposity and associated adverse metabolic disorders. Lineage tracing in mice revealed that adipose progenitor cells (APCs) in visceral fat undergo extensive adipogenesis during middle age. Single-cell RNA sequencing identified a distinct APC population, the committed preadipocyte, age-enriched (CP-A), emerging at this age, which demonstrated elevated proliferation and adipogenesis activity, via its surface leukemia inhibitory factor receptor and the classical JAK–STAT3 pathway [22]. VAT is primarily drained by the portal vein system, thus the excessive lipolytic state of hypertrophic VAT adipocytes expose the liver to high concentrations of free fatty acids and glycerol. This results in multiple hepatic metabolic disturbances, including reduced hepatic insulin clearance (leading to hyperinsulinemia), increased triglyceride-rich lipoprotein production, and enhanced hepatic glucose output. Consequently, VAT accumulation is frequently associated with glucose intolerance and type 2 diabetes.

Another manifestation of VAT dysfunction is the development of chronic low-grade inflammation. The inflammatory activation in VAT represents an early event in the aging process. While immune cells become broadly activated across multiple tissues during aging, this activation is first detectable in white adipose depots during middle age [23]. Mitochondrial damage-induced mitochondrial DNA (mtDNA) release may activate immune responses through the cGAS–STING pathway [24], thereby linking metabolic and immune processes. Targeted proteomic analysis of multiple tissues in aging mice has also revealed alterations in lipid metabolism and inflammation-related processes, specifically in WAT during aging [25]. In aged states, both mouse and human WAT show reduced relative numbers of eosinophils, increased macrophage infiltration, and elevated proinflammatory cytokine expression that correlates with systemic low-grade inflammation. Supplementation of young mouse eosinophils to aged mice can ameliorate both local adipose tissue and systemic low-grade inflammation [26]. Aging human and mouse VAT demonstrates increased γδ T cells with tissue-resident memory T cell phenotypes, and their depletion reduces VAT and systemic IL-6 levels, alleviating aging-related tissue inflammation [27]. Regulatory T cells (Tregs), which normally exhibit immunosuppressive properties and maintain insulin sensitivity by suppressing effector T cells and other immune cells in adipose tissue, paradoxically contribute to insulin resistance in aged mice [28]. In aged adipose tissue, B cells show an age-dependent increase in female mice, and their depletion improves Treg accumulation in VAT of aged mice, leading to improved immune remodeling [29]. Immunoglobulin IgG progressively accumulates in adipose tissue during early aging, activating macrophage-mediated inflammatory responses that release TGF-β. This induces fibrosis in adipocyte precursor cells, leading to adipose tissue fibrosis and ultimately impairing metabolic function. Intervention to reduce IgG accumulation significantly improves metabolic decline during aging and extends healthspan [30, 31]. Under obese conditions, IgG additionally interacts directly with insulin receptors to inhibit insulin signaling, contributing to insulin resistance and metabolic dysregulation.

### Decline in thermogenic adipose tissue function

Recent studies utilizing PET-CT and adipose tissue biopsies have identified functional brown and beige thermogenic adipose tissue in the ventral cervical and supraclavicular regions of adult humans, with a distribution pattern progressing from white to beige to brown adipocytes from superficial to deep layers [32]. Thermogenic adipose tissue plays a crucial role in non-shivering thermogenesis, dissipating excess energy as heat and promoting energy metabolism. Enhanced activity of brown and beige adipose tissue increases energy expenditure, improves glucose and lipid metabolism, and counteracts adipose dysfunction and obesity. Each gram of active brown adipose tissue can consume 50–300 kilocalories (kcal) daily, depending on activity level and ambient temperature. Under cold exposure conditions (15°C–19°C) and during recovery after local hyperthermia (41°C), thermogenic adipose activity significantly increases with marked elevation in energy expenditure. However, during aging, both the quantity and thermogenic capacity of brown and beige adipose decline, with reduced responsiveness to cold stimulation or β3-adrenergic receptor activation and impaired browning of beige adipocytes. These changes lead to compromised energy expenditure and impaired clearance of energy substrates (glucose and free fatty acids), resulting in abnormal thermoregulation and energy metabolism imbalance in elderly individuals, including lipid accumulation and glucose metabolism disorders. Therefore, age-related decline in thermogenic adipose function represents one of the driving factors for metabolic dysfunction in the elderly and accelerated progression of aging-related diseases, serving as a reliable indicator of adipose aging.

Infrared thermography has been employed to assess adipose thermogenesis under cold exposure, local hyperthermia, and caffeine stimulation. Following acute cold exposure, infrared thermography demonstrates increased skin temperature over the supraclavicular fossa and sternal regions [33]. However, the thermogenic capacity under cold exposure is significantly reduced in aged populations [34]. Furthermore, application of local heat to the interscapular adipose region results in detectable heat production in the supraclavicular fat depot via infrared thermography, indicating that local hyperthermia can induce human adipose thermogenesis [35]. Studies also show that caffeine intake leads to rapid temperature increase in the infraclavicular region and brown adipose activation, detectable by infrared thermography within short time frames [36].

Whole-room indirect calorimetry estimates metabolic rate by measuring oxygen consumption and carbon dioxide production. The respiratory quotient, calculated as the ratio of oxygen consumption to carbon dioxide production, enables determination of total energy expenditure. Metabolic chamber measurements require sophisticated instrumentation. Advanced human metabolic chambers have evolved from single-function devices, measuring not only basic metabolic indices but also physiological parameters, blood biochemical markers, and behavioral data. By modulating environmental factors, including temperature, light, and oxygen content, modern metabolic chamber platforms can create diverse simulated environments with bidirectional interaction capability, providing partial assessment of thermogenic adipose metabolic function.

### Aging of adipose tissue around vital organs, ectopic lipid deposition, and major chronic diseases

In addition to classical adipose depots, vital organs such as the heart and kidneys are surrounded by adipose tissues. The accumulation and aging of these peri-organ adipose tissues accelerate functional decline and metabolic abnormalities of vital organs. Furthermore, with the advancement of age, systemic adipose tissue aging leads to diminished lipid storage capacity, resulting in excessive free fatty acids being deposited as triglycerides in non-adipose tissues (e.g. heart, liver, pancreas, and skeletal muscle). This ectopic lipid deposition (both intracellular and intercellular) in vital organs increases the risk of metabolic syndromes, including insulin resistance, hypertriglyceridemia, hypercholesterolemia, and cardiovascular diseases in elderly individuals and those with lipid metabolism disorders. Peri-organ adipose tissue accumulation/aging and ectopic lipid deposition have been overlooked in clinical assessment and intervention, warranting greater attention in future clinical practice.

#### Pericardial adipose tissue accumulation

Pericardial adipose tissue comprises epicardial and paracardial fat. With aging, both epicardial fat volume and paracardial fat thickness increase significantly [37, 38]. Epicardial adipose tissue has been extensively demonstrated to possess metabolic activity, secreting inflammatory cytokines (e.g. TNFα, IL-6) that induce local inflammation, impair coronary endothelial function, promote atherosclerotic plaque formation and destabilization, thereby increasing coronary heart disease risk [39]. Clinical studies show significant associations between epicardial fat volume and atrial fibrillation, coronary artery disease, cardiac dysfunction, and heart failure [40, 41]. Pericoronary adipose tissue (PCAT) is epicardial fat surrounding coronary arteries. It has been demonstrated that perivascular fat attenuation index correlates with epicardial fat density and volume, collectively influencing coronary artery disease progression. Meta-analyses indicate that higher coronary fat attenuation index has prognostic value for adverse cardiovascular events [42, 43]. Additionally, myocardial fat infiltration impairs diastolic function, increases left ventricular load, and associates with cardiac hypertrophy and heart failure [39, 44, 45]. Increased paracardial fat volume also elevates heart failure risk [46]. The heart-pericardial fat interaction likely occurs through local metabolite exchange and inflammatory responses, further increasing cardiac workload. In summary, increased pericardial fat serves as an independent predictor for cardiac diseases, with pericardial fat indices significantly correlating with cardiovascular disease incidence and prognosis.

#### Perirenal adipose tissue accumulation

Excess adipose tissue can accumulate around the kidneys as perirenal and renal sinus fat. Excessive renal adipose tissue leads to renal dysfunction, lipid metabolism disorders, gluconeogenesis dysregulation, and cardiovascular impairment. Both perirenal and renal sinus fat increase with age [47, 48]. Elevated perirenal fat levels strongly correlate with renal injury and function decline, showing inverse correlation with glomerular filtration rate and positive correlation with urinary albumin excretion rate [49, 50]. In type 2 diabetes, perirenal fat thickness is a better prediction of chronic kidney disease development than total fat, subcutaneous fat, or VAT [51]. Beyond renal disease, perirenal fat thickness/volume positively associates with metabolic disorders, including type 2 diabetes, hyperuricemia, and atherosclerosis [52–54]. Notably, perirenal fat exhibits browning capacity and shows bidirectional crosstalk with renal carcinoma cells. Renal carcinoma cells can induce perirenal fat browning via PTHrP secretion, while browned perirenal fat releases lactate into tumor microenvironment, promoting renal cancer growth and metastasis [55].

#### Mesenteric adipose tissue accumulation

Mesenteric adipose tissue (MAT) abnormality (hypertrophy and wrapping) has long been recognized as a hallmark of Crohn’s disease (CD) [56]. Characteristic MAT hyperplasia occurs in CD patients [57]. Hyperplastic MAT extends from the mesenteric root to the intestinal inflammatory lesions, exhibiting creeping expansion along bowel surfaces (creeping fat, CF). As a rich source of fatty acids, cytokines, growth factors, and adipokines, CF plays crucial roles in immune and inflammatory regulation [58]. Recent studies suggest gut microbiota (e.g. *Clostridium innocuum*) translocation to mesenteric fat promotes CF formation, potentially contributing to elderly-onset inflammatory bowel disease [59].

#### Bone marrow adipose tissue accumulation

Bone marrow adipose tissue (BMAT, yellow marrow) is highly heterogeneous and primarily resides in long bones (tibia, femur, humerus), vertebrae, and iliac crest. Marrow adipogenesis represents a metabolic consequence of obesity and aging, accompanying conditions like type 1/2 diabetes, anorexia nervosa, estrogen/growth hormone deficiency, hematopoietic impairment, and osteoporosis [60]. Aging bone microenvironment favors bone resorption over formation, accompanied by BMAT accumulation [61]. Specifically, skeletal stem/stromal cells (SSCs) preferentially undergo adipogenesis rather than osteogenesis during aging. Concurrently, osteoblasts and osteocytes exhibit increased apoptosis, reduced numbers, and functional impairments, including compromised mechanosensing, intercellular regulation, and exosome secretion [62].

BMAT is broadly classified into constitutive (cMAT, growth plate-associated) and regulated (rMAT, diet/age-induced) subtypes. cMAT emerges within 1–4 weeks postpartum in distal tibia, containing large adipocytes with unsaturated lipids and high C/EBPα/β expression [63]. Conversely, rMAT adipocytes appear later, contain saturated lipids, and dynamically respond to physiological stimuli [64]. Recent data show BMAT expansion in obesity is associates with increased osteoporosis and fracture risk [65]. In healthy individuals, BMAT increases with age–lumbar vertebrae contain about 20%–30% BMAT at age 20, increasing about 7% per decade to reach 50% by age 50 [66]. Age-related BMAT expansion accompanies oxidative stress, cellular senescence, and reduced osteoprogenitor cells [67].

#### Hepatic lipid deposition

Hepatic steatosis (ectopic triglyceride deposition) drives MASLD (metabolic dysfunction-associated steatotic liver disease), MASH (metabolic dysfunction-associated steatohepatitis), fibrosis, cirrhosis, and hepatocellular carcinoma [68] . Age-related waist circumference enlargement and visceral fat expansion strongly associate with fatty liver and insulin resistance. The “two-hit” pathogenesis theory posits that hepatocellular lipid accumulation triggers cytotoxic events, inducing hepatic inflammation. VAT-derived free fatty acids and cholesterol enhance hepatic triglyceride synthesis, while insulin resistance reduces peripheral glucose uptake, promoting hepatic *de novo* lipogenesis. Lipid intermediates (diacylglycerols, ceramides) inhibit insulin signaling, creating a vicious cycle. Inflammatory cytokines/adipokines from liver and adipose tissue sustain hepatic injury and systemic low-grade inflammation [69]. Clinical studies show a 41.9% fatty liver prevalence in elderly, with decreased prevalence in advanced age that reflecting fibrosis progression [70]. Postmenopausal women, especially overweight/obese or abdominally obese individuals, exhibit higher fatty liver risk [71]. Recent findings indicate hepatic steatosis accelerates brain aging—MASLD patients aged 60–70 show 4.2-year older brains, while those < 60 show 7.3-year older brains versus controls [72].

#### Skeletal muscle lipid deposition

Skeletal muscle, comprising myofibers, satellite cells, and interstitial cells, undergoes lipid deposition (intermuscular adipocyte infiltration and intramyocellular lipids) during aging, and exhibits reduced muscle mass, strength, and endurance—key features of sarcopenia [73, 74]. Chronic fat infiltration induces fiber-type switching (fast-to-slow twitch), impairing contraction velocity and power output, which preferentially impact anaerobic performance [75, 76]. Lipid-derived fatty acids and proinflammatory cytokines induce muscle insulin resistance, delay post-injury repair, and prolong recovery [77]. Paraspinal muscle fat infiltration contributes to lumbar dysfunction, chronic back pain, and myopathy (evidenced by electromyography, elevated serum creatine kinase [CK], and lipid-laden type I fibers on biopsy) [78]. Preventing stem cell adipogenic/fibrogenic differentiation is crucial for maintaining muscle function during aging.

#### Pancreatic fat deposition

Aging pancreas undergoes atrophy, steatosis, fibrosis, inflammatory infiltration, and metaplasia [79]. Pancreatic fat deposition impairs β-cell function, causing insulin secretion defects (diabetes, impaired fasting glucose, glucose intolerance), while increasing pancreatitis and pancreatic cancer risk [80]. A Chinese study (*n* = 8097) reported 16% pancreatic steatosis prevalence, with advanced age, obesity, diabetes, and non-alcoholic fatty liver disease (NAFLD) as independent risk factors [81]. Peripancreatic fat volume independently predicts acute pancreatitis, pancreatic pseudocysts, and systemic inflammatory response [82]. During pancreatitis, peripancreatic fat necrosis (via lipase-mediated fatty acid release) causes adjacent parenchymal damage [83]. Intralobular fat infiltration and fibrosis independently predict pancreatic intraepithelial neoplasia, suggesting that pancreatic steatosis may promote carcinogenesis [84, 85]. Pancreatic cancer patients with steatosis exhibit more lymph node metastasis and shorter survival, indicating that pancreatic fat deposition may accelerate cancer progression [86].

### Recommendations

(1) BMI, waist circumference, body fat content and distribution may serve as functional biomarkers for predicting adipose aging (Class A evidence, Level IIa recommendation).
(2) Decreased core body temperature and local thermogenic capacity under cold exposure may serve as functional biomarkers for predicting adipose aging (Class B evidence, Level IIb recommendation).
(3) Ectopic lipid deposition, including hepatic steatosis, skeletal muscle lipid accumulation, and pancreatic fat deposition, may serve as functional biomarkers for predicting adipose aging (Class B evidence, Level IIb recommendation).

## Imaging markers

Clinical imaging has been utilized to assess distribution, quantity, and quality of various types of fat tissues, including subcutaneous fat, visceral fat, pericardial fat, renal pericardial fat, and bone marrow fat. Imaging can provide important clues for the diagnosis, evaluation, and treatment of obesity, metabolic diseases, cardiovascular diseases, kidney diseases, and other related diseases. Currently available imaging techniques for adipose measurement include ultrasound (US), dual-energy X-ray absorptiometry (DEXA), computed tomography (CT), and magnetic resonance imaging (MRI).

### Visceral and subcutaneous adipose tissue assessment

In a study of large-scale Asian populations, visceral adiposity is measured by DEXA to calculate the visceral fat index. It has been found that a higher visceral fat index is associated with type 2 diabetes and other metabolic diseases [87]. Additionally, the DEXA results show a positive correlation between visceral adiposity and cardiovascular metabolic risk factors, as well as a negative correlation with brain cortical thickness and cognitive function [7, 8, 88, 89] .

MRI and CT are considered the gold standards for quantitatively measuring VAT. An intra-abdominal fat area of ≥ 100 cm² has been widely accepted as a criterion for defining visceral obesity in East Asian populations [90]. VAT volume under CT quantification has been proven to be an independent risk factor for the occurrence of metabolic-related diseases [91]. The Chinese visceral fat index (CVAI), developed through abdominal VAT CT, has been shown to be closely related to visceral obesity and higher HOMA-IR, demonstrating considerable potential for assessing metabolic risk [91]. This index has been validated in several Chinese cohorts, showing strong associations with the risk of cardiovascular diseases, diabetic complications, liver diseases, and other metabolic-related conditions [92–95].

In addition to assessing visceral adiposity, imaging techniques can also detect the texture features of VAT. Combined with machine learning techniques, novel imaging biomarkers RunEntropy was established using CT scans of VAT texture features, which show a significant association with the incidence of insulin resistance and metabolic syndrome [96].

The thickness of subcutaneous fat in several body areas, such as the abdomen, buttocks, legs, and upper arms, can be assessed by ultrasound or MRI. In a study in Japan, ultrasound was used to measure the subcutaneous fat thickness at various body sites, including the right cheek, chin, chest, abdomen, iliac crest, triceps, subscapular area, back, thigh, knee, and calf. The results showed that subcutaneous fat accumulation progressed toward the central body with the increase of age [97]. Similarly, assessment of subcutaneous fat thickness using MRI in different age groups of women revealed a decrease in subcutaneous fat thickness with age [98], which is associated with a higher risk of hip fractures in the elderly [99]. MRI has been used to comprehensively assess the relationship between subcutaneous fat thickness and all-cause mortality, which revealed a significant negative correlation independent of BMI. This correlation is not confined to the elderly population but is also evident in younger and middle-aged individuals [100].

Blood flow plays an important role in regulating adipose tissue function. Imaging by [^15^O]-H₂O PET-CT and skin Doppler ultrasound (i.e. color Doppler flow imaging, CDFI) are capable of providing an assessment of blood flow [101]. The measurement of adipose tissue blood flow combining MRI and CT scans revealed that blood flow in VAT or SAT fat depots is positively correlated with insulin sensitivity [102]. In subcutaneous fat, PET-CT has also shown that blood flow is a decisive factor for fatty acid uptake in subcutaneous fat [103]. Notably, most imaging studies on VAT have focused on populations with obesity or metabolic disease and is still insufficient in the aging populations.

### Brown fat volume assessment

Infrared thermography is a convenient and accurate instrument for assessing the thermogenic capacity of brown fat. It utilizes infrared imaging to quantitatively assess brown adipose thermogenesis under various stimulating conditions, such as cold, hyperthermia, diet, exercise, and specific interventions [33–36]. Particularly, it has been found that the thermogenic capacity of brown fat measured by infrared thermography is significantly reduced in the elderly population under cold exposure, making it a potential rapid and simple imaging method for assessing brown fat aging [34].

Currently, the gold standard for detecting brown fat activity in clinical practice is [^18^F] fluorodeoxyglucose positron emission tomography and computed tomography (^18^F-FDG-PET/CT), which quantifies brown fat activity by measuring the uptake of glucose, and is the most commonly used and well-established technique [104]. In 2016, Chen et al. published the Brown Fat Imaging Check Report Standard (BARCIST 1.0), proposing that for diagnosis of brown fat in ^18^F-FDG-labeled PET images, the standardized uptake value (SUV) of brown fat normalized to lean body mass should be ≥ 1.2, and the CT value should be within the range of −190 to −10 HU. This standard has been supported by other studies. Using ^18^F-FDG-PET/CT, researchers have revealed significant differences in brown fat volume between young and elderly individuals, with a notable decrease in elderly males [105]. However, the ionizing radiation of ^18^F-FDG-PET/CT makes it unsuitable for widespread clinical use and follow-up observations. Recently, a non-invasive metabolic MRI method based on creatine chemical exchange saturation transfer contrast (CrCEST MRI) has been developed to assess the activity of brown adipose tissue (BAT) in rodents and humans. CrCEST MRI can detect cold-induced BAT activation in humans, and its results are consistent with those measured by ^18^F-FDG PET/CT imaging, thus representing a promising non-invasive, radiation-free imaging method for brown fat activity [106].

At present, most imaging methods for assessing brown fat mass are highly dependent on factors, such as temperature, hormones, diet, drugs, and sympathetic nervous system status. However, brown fat mass is the actual weight of the tissue, which remains consistent regardless of stimulation. Therefore, accurate assessment of brown fat quality poses a significant challenge. It has been reported that ^18^F-F-DA, a dopamine analog, can visualize brown fat tissue under thermal neutral conditions [107]. Based on chemical shift encoding, MRI can detect both active and inactive brown fat tissues. The MRI-derived fat signal fraction of active brown fat is significantly lower than that of inactive brown fat, which is also reflected in the significant increase in Hounsfield units [108].

### Assessment of adipose tissue around/in vital organs

#### Epicardial adipose tissue

Epicardial adipose tissue (EAT) is located between the myocardium and the visceral layer of the pericardium, covering approximately 80% of the heart surface. It has both mechanical protective and metabolic regulatory functions. Its volume and functional abnormalities are closely related to cardiovascular diseases such as coronary artery disease and atrial fibrillation, as well as poor prognosis and heart failure in the elderly population [109, 110] . Currently, various imaging techniques, such as two-dimensional echocardiography, CT, and MRI, can be used to clinically assess EAT content [39]. Two-dimensional echocardiography can visualize and measure the thickness of EAT. In two-dimensional echocardiography, EAT is typically identified as an anechoic area between the outer wall of the myocardium and the visceral layer of the pericardium. However, in cases of inflammation or when large amounts of EAT are presented, it may appear as a dense echo area [39]. Echocardiographic measurements revealed that increased EAT thickness leads to a decrease in overall cardiac longitudinal strain, reduced left atrial contraction strain, and increased risk of heart failure, indicating that EAT thickness is independently associated with subclinical cardiac dysfunction [111].

Cardiac multidetector spiral CT and MRI can measure the volume of EAT in deeper areas that cannot be detected by transthoracic echocardiography to reflect the functional state of EAT. EAT volume measured by CT has been found to be related to myocardial fat infiltration, decreased myocardial electrical signal conduction velocity, increased electrocardiographic fragmentation, fibrosis degree, atrial conduction heterogeneity, and so on [112]. In addition, a study using coronary artery computed tomography angiography (CCTA) with a non-enhanced imaging sequence and semi-automatic methods measured the volume and density of EAT and found that a smaller EAT volume and higher EAT density are related to the occurrence of heart failure with improved ejection fraction, which suggest that reducing the volume of EAT and improving its quality may have beneficial effects on myocardial function recovery in patients with heart failure [41]. Another study using CT to measure EAT volume found that for systolic function, an increase of one standard deviation (SD) in EAT volume is associated with an increase of 0.76 mm in left ventricular end-diastolic dimension, an increase of 0.66 mm in left ventricular end-systolic dimension, and a decrease of 0.56% in left ventricular ejection fraction. In addition, larger EAT volume is associated with heart failure [40]. Notably, studies have shown that EAT volume also increases with age according to MRI assessments [109, 113].

#### Perirenal adipose tissue assessment

The adipose tissue around and inside the kidneys can be quantified non-invasively through imaging methods such as ultrasound, computed tomography, and MRI. Some clinical studies suggest that perirenal fat, renal sinus fat, and intrarenal fat may play a potential role in the development of chronic kidney disease related to obesity, insulin resistance, hypertension, and atherosclerosis [114]. Ultrasound measurement of perirenal fat thickness is negatively correlated with glomerular filtration rate (GFR) in diabetic patients [115]. The perirenal fat thickness measured by CT predicts the occurrence of chronic kidney disease (CKD) in type 2 diabetes patients. Compared with total body fat, subcutaneous or visceral fat, perirenal fat thickness can independently predict the occurrence of CKD in type 2 diabetes patients with higher efficiency, suggesting it as a superior predictive value [51]. MRI found that perirenal fat thickness is negatively correlated with the reduction of renal GFR, as well as the increase of renal vascular resistance (RVR) and renal arterial resistance (RA), suggesting that perirenal fat may affect renal blood flow dynamics and lead to renal dysfunction in type 2 diabetes patients [49]. Although perirenal fat is closely related to metabolic diseases, there are limited research focuses on aging population, which warrants further exploration.

#### Mesenteric adipose tissue assessment

Most studies use CT to quantify intra-abdominal fat area (VFA) at the third lumbar vertebra (L3) level to reflect the degree of mesenteric hypertrophy. A prospective study using MRI to detect abdominal fat thickness before and after weight loss surgery found that lower baseline mesenteric fat thickness is related to significant weight loss and metabolic syndrome relief [116]. Using 1.5-T frequency of 40–70 Hz magnetic resonance elastography (MRE) detection, it was found that the average shear wave velocity of the contralateral peristomal mesentery in Crohn’s disease (CD) patients is 7% lower than that of healthy controls [117]. In a retrospective cohort, the mesenteric crawling fat index (MCFI) was used to distinguish the severity of fibrosis and mild fibrous stenosis. The receiver operating characteristic (ROC) curve determined that, using MCFI > 3 as threshold, MCFI can accurately distinguish the degree of fibrous stenosis in the cohort [118]. In addition, the hypertrophic mesentery fat in CD patients wraps around the intestinal inflammatory site, which further exacerbates intestinal stenosis and fibrosis [59]. The relationship between mesenteric fat thickness and aging-related diseases requires further evaluation.

#### Bone marrow adipose tissue assessment

Bone marrow adipose tissue (BMAT) is a highly dynamic tissue that plays a vital role in various physiological and pathological processes, including osteoporosis and bone tumors. Studies in rodents and humans have confirmed that aging is associated with a significant increase in BMAT volume, while bone density decreases [119]. The bone marrow fat ratio refers to the proportion of bone marrow fat in the total volume of bone marrow, which can be quantified by MRI.

The bone marrow fat ratio of the spine, pelvis, ribs, and limbs can be measured using a 1.5-T MRI device, based on multi-gradient echo chemical shift encoding water-fat imaging or T1 Dixon imaging. The results showed that bone marrow fat ratio of the spine or trunk bones is correlated to age, which can be used as a potential indicator of bone aging. In patients with normal bone marrow, the BMAT content measured by magnetic resonance spectroscopy (MRS) increases with age. The content of BMAT in patients over 61 years old is between 50% and 80% [120]. Assessment and comparison of vertebral BMAT and volume bone density (vBMD) showed that in men, vertebral BMAT gradually increased throughout life, while in women, BMAT increased sharply between the ages of 41 and 60, exceeding that of men. vBMD gradually decreased with age in men, while in women, vBMD sharply decreased after the age of 40, indicates significant age and gender differences in the lumbar BMAT and vBMD [121].

Bone marrow fat ratio increase may be related to osteoporosis in the elderly and metabolic diseases such as diabetes and obesity. BMAT can be used as a diagnostic marker and potential therapeutic target for osteoporosis [62]. Recently, a new deep learning method using data from the British Biobank Dixon MRI for large-scale bone marrow adiposity analysis was reported. The results showed that the model accuracy is equal to or exceeds the traditional U-Net, with a Dice score of 91.2% (spine), 94.5% (femoral head), 91.2% (total hip joint), and 86.6% (femoral shaft), thereby generated the expected relationships between the bone marrow fat fraction and age, gender, and bone density. Moreover, the new site- and gender-specific characteristics have been identified in this study [122].

#### Hepatic fat accumulation assessment

Liver biopsy is still the gold standard for the diagnosis of fatty liver. However, the invasiveness of liver biopsy and the risk of complications such as bleeding limit its clinical application. Moreover, the liver tissue sample obtained by liver biopsy only represents 1/50,000 of the liver volume. Given the spatial heterogeneity of diffuse liver disease and the uneven distribution of steatosis within the liver parenchyma, limited liver sampling can also lead to errors in diagnosis and disease staging. Therefore, non-invasive examinations, including ultrasound, CT, transient elastography Fibroscan, and MRI, are still the main means of fatty liver diagnosis in clinical practice.

Ultrasound (B-ultrasound) remains the most widely used detection method due to its simplicity and speed of operation. However, it is a non-quantitative method and has poor sensitivity towards mild hepatic steatosis. When the degree of hepatocyte steatosis is less than one-third, ultrasound cannot show the characteristic changes of fatty liver, which confounds diagnosis [123]. CT is a semi-quantitative measurement for the degree of fatty liver disease (FLD) using the spleen density value as reference. However, similar to ultrasound, the diagnostic performance of CT is poorer when the degree of steatosis is lighter. In cases of mild steatosis with fat content of 10%–20%, the sensitivity of CT diagnosis is only 52%–62% [124]. Transient elastography (FibroScan) is characterized by its simplicity, speed, ease of operation, good safety, and tolerability. It quantitatively detects the degree of hepatic steatosis through the controlled attenuation parameter (CAP) based on the feature of significant attenuation of ultrasound in fat tissues. CAP is a novel and promising non-invasive detection technology for fatty liver, which is more sensitive than ultrasound and CT. It can accurately detect fatty liver with hepatic steatosis greater than 5%. Moreover, the CAP value reflecting hepatic steatosis is not affected by the etiology of liver disease [125]. Compared with liver biopsy, CAP is less affected by sampling error because its detection area is 100 times larger than that of liver biopsy [126]. The CAP value is closely related to fatty liver and its underlying etiology (obesity, dysmetabolism of glucose and lipids, and metabolic syndrome). To some extent, changes in the CAP value during follow-up can reflect the improvement or progression of hepatic steatosis and metabolic disorders [127].

Magnetic resonance imaging proton density fat fraction (MRI-PDFF) can quantitatively analyze the lipid content in liver tissue by calculating the ratio of the mobile proton density derived from triglycerides in the liver to the total mobile proton density of triglycerides and water. It is considered the gold standard for measuring hepatic lipid content [128]. When using a threshold of 6.4%, MRI-PDFF has a sensitivity of 86% and a specificity of 83% for diagnosing grade 1 steatosis (defined as < 33% of parenchymal cells being infiltrated by lipid droplets), showing good correlation with histopathological diagnosis. In a clinical analysis involving 1100 patients with chronic liver disease, MRI-PDFF was used to measure hepatic fat content and predict hepatic steatosis graded as G1, G2, and G3, with an area under curve (AUC) ranging from 0.91 to 0.98, demonstrating significant correlation with liver biopsy [129]. In a clinical study of individuals with moderate abdominal obesity, MRI-PDFF was used to assess the percentage of intrahepatic fat (%IHF), abdominal fat depots, and fat percentages in the pancreas and renal sinus. The study evaluated the relationships between hepatic lipids and abdominal fat depots, pancreatic and renal sinus fat, as well as their links with metabolic state biomarkers. After adjusting for age, gender, waist circumference, and VAT, %IHF was found to be associated with elevated levels of liver enzymes, blood glucose, lipids, and inflammatory biomarkers. Even within the normal range of %IHF < 5%, %IHF was correlated with specific risk factors, indicating that an increase in %IHF constitutes a continuous risk factor for metabolic status [130]. In a large-scale population study, MRI revealed a median proton density fat fraction (PDFF) of 3.9% (range: 0.6%–41.5%) in the population. Among males aged 20–50 years, hepatic lipid content increased with age. In females, hepatic lipid content remained relatively stable before the age of 40 and then increased continuously between the ages of 40 and 65 [131].

#### Skeletal muscle fat accumulation assessment

Assessment of muscle lipid deposition using CT primarily relies on muscle radiodensity. Muscle radiodensity decreases with increasing muscle lipid content, making it possible to assess muscle lipid deposition using mean muscle radiodensity. Additionally, recent studies have proposed regional segmentation of intermuscular adipose tissue (IMAT) to evaluate muscle fat infiltration [132]. Based on this, a muscle mass map can be constructed to display segmented areas of IMAT, low attenuation muscle areas (LAMA), and normal attenuation muscle areas (NAMA), further illustrating the distribution of muscle mass. Research analysis has shown that the mean value of the NAMA index decreases with age, while the indices and mean values of LAMA and IMAT increase with age [133]. In a recent prospective cohort study, the cross-sectional area of skeletal muscle and IMAT were measured at the level of T12 vertebra. The skeletal muscle index (SMI) was calculated by dividing the skeletal muscle area by the square of height, and the results showed that SMI is negatively correlated with age, while IMAT is positively correlated with age [134].

#### Pancreatic fat accumulation assessment

Pancreatic fat deposition (PFD) is closely related to various diseases. Early intervention and reduction of fat deposition may help prevent and reverse diseases caused by PFD. Therefore, accurate measurement of pancreatic fat content (PFC) is particularly important. Histopathology is the gold standard for diagnosing PFD. However, due to the invasive nature and high risk of percutaneous biopsy, its application is limited. In clinical practice, abdominal ultrasound, CT, and MRI are commonly used to quantitatively measure PFC [135–137]. Ultrasound is highly subjective and easily affected by tissue interference, often resulting in imprecise measurements. Studies have shown that quantitative imaging based on CT can accurately predict pancreatic fat infiltration. Some studies suggest a pancreatic fat threshold of 36 HU [138]. In other studies, CT is commonly used for semi-quantitative assessment of pancreatic fat in the form of the pancreas–spleen CT value difference (P–S) and pancreas–spleen CT value ratio (P/S). A cross-sectional study from China using ROC curves calculated the critical values of P/S and P–S as ≤ 0.72 and ≤−13.33, respectively [139]. MRI results have also been shown to have high accuracy and reproducibility and are significantly correlated with histologically assessed fat content. However, it is time-consuming and expensive. Currently, there is no standard grading system for the severity of fatty pancreas. In a cross-sectional study with histological specimens, pancreatic fat was divided into three grades: mild (fat infiltration < 10% of total pancreatic tissue), moderate (fat infiltration 10%–20% of total pancreatic tissue), and severe (fat infiltration > 20% of total pancreatic tissue) [140]. At present, the use of magnetic resonance fat quantification with least squares estimation and iterative decomposition of water and fat imaging with asymmetric echoes (IDEAL-IQ) is the most accurate technique for measuring PFC. Clinical studies have reported an association between pancreatic fat infiltration and impaired β-cell function in obese patients, suggesting that increased pancreatic fat may be a potential risk factor for the development of type 2 diabetes. By measuring the pancreatic fat fraction (PFF) using MRI IDEAL-IQ, PFF greater than 6.2% is defined as pancreatic fat infiltration [141]. A recent clinical study showed that, using MRI IDEAL-IQ to measure the PDFF of the pancreas, PDFF > 12.1% in the pancreatic body can be used as a diagnostic marker for severe pancreatitis [142].

### Recommendations

(1) Visceral and subcutaneous adipose tissue may serve as imaging biomarkers of adipose aging. An increase in visceral fat volume and a decrease in subcutaneous fat thickness can help predict the state of adipose aging. Assessment can be performed using US, DEXA, MRI, and CT (Level A, Class Ia).
(2) Brown adipose tissue can serve as an imaging biomarker of adipose aging. A reduction in brown fat volume and a decrease in thermogenesis can help predict the state of adipose aging. Assessment can be performed using infrared thermography (Level A, Class Ia).
(3) Decreased brown adipose tissue activity can serve as an imaging biomarker of adipose aging. A decline in glucose uptake by brown fat can help predict the state of adipose aging. Assessment can be performed using ^18^F-FDG-PET/CT or CrCEST MRI (Level A, Class Ia).
(4) Adipose tissue blood flow can serve as an imaging biomarker of adipose aging. A reduction in blood flow can help predict the state of adipose aging. Assessment can be performed using Doppler ultrasound or PET-CT (Level A, Class Ia).
(5) Epicardial adipose tissue can serve as an imaging biomarker of adipose aging. An increase in epicardial fat volume can help predict its aging state. Assessment can be performed using two-dimensional echocardiography, MRI, and CT (Level B, Class IIb).
(6) Perirenal adipose tissue can serve as an imaging biomarker of adipose aging. An increase in perirenal fat thickness can help predict its aging state. Assessment can be performed using MRI and CT (Level B, Class IIb).
(7) Mesenteric fat can serve as an imaging biomarker of adipose aging. An increase in mesenteric fat thickness can help predict its aging state. Assessment can be performed using MRE and CT (Level B, Class IIb).
(8) Bone marrow adipose tissue can serve as an imaging biomarker of adipose aging. An increase in bone marrow fat ratio can help predict its aging state. Assessment can be performed using MRI and CT (Level B, Class IIb).
(9) Ectopic hepatic lipid deposition can serve as an imaging biomarker of adipose aging. An increase in the degree of hepatic steatosis can help predict the functional aging state of adipose tissue. Assessment can be performed using biopsy, ultrasound, CT, and MRI-PDFF (Level B, Class IIb).
(10) Ectopic skeletal muscle lipid deposition can serve as an imaging biomarker of adipose aging. A decrease in mean muscle radiodensity can help predict its aging state. Assessment can be performed using CT (Level B, Class IIb).
(11) Ectopic pancreatic lipid deposition can serve as an imaging biomarker of adipose aging. An increase in pancreatic fat content can help predict its aging state. Assessment can be performed using CT, and MRI (Level B, Class IIb).

## Humoral and biopsy markers

Blood and urine are the main components of body fluids. Due to their non-invasive or minimally invasive nature, high sensitivity, and ease of accurate measurement, they may become important biological markers for assessing adipose aging, which could be measured by mass spectrometry or enzyme-linked immunosorbent assay (ELISA). Adipose tissue biopsy is relatively easy and safe to obtain compared to other tissues, and can provide a wealth of molecular information for precise quantification and digitization. Combined with artificial intelligence (AI), adipose tissue biopsy will play an increasingly important role in evaluating adipose tissue aging. This consensus aims to recommend adipose aging-related markers, and the identification of blood, urine, and adipose biopsy tissue markers may provide biological markers highly correlated with the level of adipose aging. Therefore, this consensus focuses on aging markers with disease predictive significance. Adipose tissue aging markers change accordingly in distinct metabolic diseases, while blood and gut microbiota are closely related to other organ lesions. Thus, in cohort studies using adipose aging markers, care should be taken to decipher the impact of these factors.

### Adipocytokines

Adipose tissue can secrete a large number of bioactive molecules called adipokines, and the dysregulation of adipokine biosynthesis and secretion is considered a key feature of obesity and age-related diseases. Among them, leptin is a classic adipokine that can communicate with the central nervous system to regulate appetite, satiety, and energy expenditure. Leptin, especially leptin bioavailability, is associated with a reduced incidence of dementia and Alzheimer’s disease (AD). Serum leptin levels are positively correlated with body fat content, and elderly individuals often experience leptin resistance, that is, with increasing age, serum leptin levels increase, but the central nervous system’s responsiveness to leptin decreases, which may have a negative impact on cognitive function [143, 144]. Adiponectin is an adipocytokine with anti-inflammatory and insulin-sensitizing effects. It can alleviate inflammation and improve metabolic status by blocking NF-κB activation and inhibiting the synthesis of pro-inflammatory cytokines, and is positively correlated with longevity. Adiponectin may also improve neuron metabolism, muscle function, and cardiovascular health through adiponectin receptors. For example, studies have reported that centenarians have higher levels of adiponectin, which may be related to extended lifespan [145]. Currently, a novel biomarker for assessing adipose tissue dysfunction is the adiponectin: leptin (AL) ratio. A low AL ratio indicates adipose tissue dysfunction and an increased risk of metabolic-related diseases. An ancillary study investigated 163 obese elderly individuals living in the community and found that the AL ratio showed significant negative correlation with BMI, waist circumference, and serum insulin levels. After exercise and weight loss interventions, the AL ratio increased significantly from baseline levels [146].

Resistin is a fat tissue-derived secretory factor that can lead to increased low-density lipoprotein (LDL) levels and cause inflammatory responses. Resistin is also secreted by immune cells such as monocytes, macrophages, and bone marrow cells. Resistin can promote inflammation, insulin resistance, and atherosclerosis [147]. Studies have shown that resistin levels are higher in the elderly and are associated with age-related chronic diseases such as inflammation and insulin resistance [148], suggesting that blood resistin levels may serve as a potential marker of adipose aging.

Retinol-binding protein-4 (RBP-4), fibroblast growth factor-21 (FGF-21), and adiponectin are effective biomarkers for a variety of cardiovascular and metabolic diseases [149] . A cohort study conducted over 5 years in the Chinese population showed that incorporating RBP-4, FGF-21, and adiponectin was more accurate in predicting steatotic liver disease (SLD) than traditional models [150]. Considering that RBP4 and FGF-21 have been reported to be expressed and play a role in metabolic regulation in multiple vital metabolic organs, such as the liver and adipose tissue, they may serve as potential adipose aging markers.

### Metabolites

Studies have found that creatine in human adipose tissue shows a significant negative correlation with multiple obesity indicators and insulin resistance, while ceramide C18:1-Cer levels are positively correlated with obesity indicators [151]. Non-targeted metabolomics analysis of abdominal SAT in obese and non-obese women revealed that glutamine content in adipose tissue was significantly reduced in obese individuals, which promoted inflammation in adipose tissue [152]. A study using metabolomics to detect visceral fat metabolites found that in pathologically obese patients, the levels of oxidative stress markers, such as glycerophosphocholine, glycerophosphoethanolamine, glycerophosphoserine, ceramides, sphingolipids, and phospholipid precursors in visceral fat were significantly increased [153]. Another study used serum metabolomics to identify and validate metabolite markers related to visceral fat in two independent cohorts, and found that amino acids (such as leucine, isoleucine, glutamine, etc.), organic acids (such as lactic acid), and lipoprotein subclasses (such as HDL, very low-density lipoprotein [VLDL], etc.) in serum were significantly correlated with visceral fat volume [154]. Animal studies have shown that elevated levels of histamine [155] and ceramide [156] in brown adipose tissue of aged mice are potential biomarkers. During the aging process, PRDM16-expressing adipocytes secrete β-hydroxybutyrate (BHB), which inhibits fiber formation in precursor and promotes beige fat differentiation. Dietary supplementation of BHB in aged mice also reduces fat fibrosis and promotes beige fat formation [157]. During the aging process, the gradual accumulation of ceramides synthesized by adipocytes inhibits thermogenic fat metabolism [158]. Inhibiting ceramide synthesis in thermogenic fat cells can improve adipose tissue function. In a longevity mouse model (Ame dwarf mice, df/df), an ω-3 fatty acid metabolite, 5-hydroxyeicosapentaenoic acid (5-HEPE), was found to be increased in BAT and blood, leading to increased thermogenic capacity and insulin sensitivity in df/df mice. This metabolite is positively correlated with BAT activation in humans and negatively correlated with body weight, insulin resistance, and triglyceride levels [159]. These metabolites are promising as specific markers for assessing the level of adipose aging, but there is currently a lack of relevant clinical evidence. They can be considered as candidate markers for validation in subsequent cohort studies.

### Urine markers

8-oxo-guanine and 8-oxo-deoxyguanosine are the main products of RNA and DNA oxidative stress in the body, respectively and are biomarkers reflecting oxidative stress. In a cohort of 1128 healthy Chinese individuals, it was found that the levels of 8-oxo-guanine and 8-oxo-deoxyguanosine in urine increased with age [160]. In elderly individuals with cardiovascular disease and type 2 diabetes patients, the level of 8-oxo-guanine in urine was positively correlated with the frailty index, as well as with age-related diseases and mortality [161–163]. Whether their origin is mainly from adipose tissue, and whether they can be used for early warning of adipose tissue aging, still requires further study.

8-isoprostane is a class of non-enzymatic eicosanoids produced by the peroxidation of arachidonic acid by oxygen free radicals and can reflect the level of lipid peroxidation in the body. Both aging and obesity are associated with increased levels of plasma 8-isoprostane [164]. A study also showed that urinary 8-isoprostane levels increased in obese patients and further increased in metabolically unhealthy obese individuals [21]. In the elderly population, higher urinary 8-isoprostane levels were linked to the development of type 2 diabetes [165], indicating that increased urinary 8-isoprostane levels may be associated with adipose tissue aging and metabolic disorders, suggesting that increased urinary 8-isoprostane levels may be used to reflect adipose tissue aging.

Prostaglandin E-M (PGE-M) is a stable end metabolite of prostaglandin E2 (PGE2), an inflammatory mediator that is unstable in the body and is rapidly metabolized into stable PGE-M via prostaglandin 15 dehydrogenase and excreted in urine. Therefore, urinary PGE-M levels are used to assess the overall level of PGE2 production in the body. In the Rotterdam Study, it was found that urinary PGE-M levels increased with age and were associated with the risk of cardiovascular death [166]. In HIV patients or breast cancer patients, urinary PGE-M levels were also found to be positively correlated with aging, BMI, and increased visceral fat tissue [167, 168]. Therefore, urinary PGE-M may also be a biomarker reflecting adipose tissue aging.

### Recommendations

(1) Serum adipose tissue-derived proteins such as leptin, adiponectin, and resistin can be considered as markers for predicting adipose aging, and their expression changes suggest the possibility of adipose aging (Level A, Class Ia).
(2) Serum metabolites such as creatine and ceramides can be considered as markers for predicting adipose aging, and their concentration changes suggest the possibility of adipose aging (Level B, Class IIb).
(3) Urine markers, such as 8-oxo-guanine, 8-oxo-deoxyguanosine, and PGE-M, can be considered as markers for predicting adipose aging, and their level changes suggest the possibility of adipose aging (Level B, Class IIb).

## Perspectives on adipose tissue aging assessment, prediction model establishment, and future standard

Adipose tissue aging involves comprehensive changes in adipose tissue from the molecular to the functional level. Therefore, to accurately evaluate the state of adipose tissue aging, it is necessary to integrate multidimensional and multiscale information. In recent years, researches exploring the biological age of adipose tissue are still in its infancy, and there is an urgent need to reach a consensus on adipose aging to promote progress in this field. Measuring the biological age of human adipose tissue and developing effective intervention strategies for adipose tissue aging is of great significance.

To answer the fundamental scientific question of “how old is the adipose tissue,” molecular and pathological detection of adipose tissue biopsies may be the best method. In the future, it is necessary to improve non-invasive, low-risk, and clinically valuable biopsy sampling detection methods and techniques. Adipose tissue biopsy can provide rich high-throughput sequencing resources and accurate adipose aging information. Adipose tissue biopsy can directly reflect the state of adipose tissue aging through SA-β-Gal staining. A prospective cohort study on the subcutaneous and omental adipose tissue biopsies from 227 severely obese patients showed that SAT aging evaluated by SA-β-Gal activity was significantly correlated with glucose metabolism abnormalities and changes in fat mass distribution [169].

In addition to SA-β-Gal activity, which reflects the degree of aging, it has been found that aging fat exhibits a series of characteristics in transcriptional features, subpopulation distribution, metabolites, epigenetic remodeling, and secreted factors. For example, with aging, adipose tissue exhibits an inflammatory phenotype, which is very similar to obesity. In a transcriptomic study of VAT in obese individuals, it was found that VAT in obese individuals exhibited a more pronounced inflammatory phenotype, and this inflammatory phenotype was even more pronounced in obese individuals with metabolic abnormalities [170]. Another study using the GTEx database of human VAT transcriptomic data also found that with age, the expression of inflammation-related secreted proteins in VAT increased [171]. The increase in inflammation in VAT may be caused by the accumulation of γδ T cells, dendritic cells, and other immune cells [27, 172].

Recently, a clinical study collected subcutaneous fat samples from young and old individuals and performed single-cell transcriptomic sequencing, identifying a subpopulation of adipose progenitor cells marked by PLAU, which significantly increased in number in old individuals and exhibited pro-inflammatory functions [173]. In high-fat diet (HFD)-induced obese mice, single-cell transcriptomic sequencing of VAT identified a cluster of cells with high p21 expression, which may mediate insulin resistance caused by adipose aging [174].

Adipose tissue is actively involved in metabolic activities and is closely related to systemic metabolic balance. However, during aging, metabolic balance is disrupted. Studies have found that catecholamine-stimulated lipolysis in SAT of women decreases with age, which may be due to the upregulation of catecholamine degradation pathways [175]. In snRNA-seq of human and mouse beige fat, high UCP1-expressing thermogenic fat cells and new metabolic pathways, such as creatine kinase, were discovered [176]. A cross-sectional study showed that in WAT, the expression levels of *UCP1* and *PRDM16* genes decreased with age, and their correlation with age was greater than that with body mass index, reflecting a decline in catabolism and thermogenic capacity [177]. Another study found that the lipid metabolism core regulator SREBP1c plays an important role in visceral adipose aging caused by obesity. The absence of SREBP1c accelerates visceral adipose aging, leading to increased inflammation in visceral fat and insulin resistance [178]. In a transcriptome-wide association study (TWAS) combined with a large-scale genome-wide association study of type 2 diabetes and single-cell RNA-seq, it was found that genes, such as *PABPC4*, *CCNE2*, *HAUS6*, *CWF19L1*, and *CCDC92* in human VAT adipocytes, may have a causal relationship with type 2 diabetes [179]. These genes may also be related to adipose tissue aging.

Like most aging tissues, adipose tissue also undergoes epigenetic changes during aging. During the differentiation of adipose stem cells into mature adipocytes, DNA methylation patterns undergo dynamic changes. During aging, the promoter regions of pro-differentiation genes (such as *PPARγ* and *C/EBPα*) undergo abnormal methylation, leading to a decline in adipocyte regeneration capacity and an increase in adipose tissue fibrosis. Histone acetylation (such as H3K27ac) and deacetylation (regulated by HDACs) affect adipocyte differentiation and lipid metabolism. SIRT1 activity decreases with age, leading to abnormal histone acetylation levels, inhibiting lipolysis and promoting inflammation [180]. The GOTO (Growing Old Together) study found that in aging individuals, adipose tissue undergoes CpG island hypermethylation, which is associated with changes in insulin sensitivity gene expression and improvements in health parameters [181].

A growing number of aging markers have been identified in adipose tissue, which are closely involved in the deterioration of adipose tissue health during aging. IgG, the main antibody in the body, increases in blood in aging humans and mice, and features a specific high accumulation in adipose tissue. Results show that IgG accumulates in the epididymal WAT of aging mice and in the pericardial fat of elderly individuals. Moreover, the accumulation of IgG in adipose tissue begins earlier, long before the appearance of aging phenotypes. IgG promotes adipose tissue fibrosis and inflammation, leading to metabolic disorders, suggesting that IgG may be an important driver of adipose tissue aging [30]. During the aging process, it was found that epididymal WAT is the main source of the extracellular matrix protein osteopontin (OPN). The aging process is accompanied by upregulated OPN expression in epididymal WAT, which increases circulating OPN levels and further promotes myocardial fibrosis, leading to myocardial dysfunction [182]. In aging mice, it was also found that Crtc2 expression in epididymal WAT significantly increased with aging. Crtc2 exacerbates metabolic disorders and inflammation by inhibiting BCAA catabolism [183].

It should be noted that some of the above research results come from animal models and need to be verified in humans. Currently, data obtained from human studies are mostly from specific populations and need to be further confirmed and unified in larger samples, multi-center, and different populations. Despite a large body of animal evidence indicating that adipose aging promotes systemic metabolic disorders and accelerates the aging process, the exact role of this mechanism in humans remains to be systematically verified. A key bottleneck currently limiting research progress is the low prevalence of human adipose tissue biopsies in clinical practice. Therefore, there is an urgent need to explore and promote more feasible sampling strategies, such as promoting routine sampling during physical examinations or implementing standardized sampling methods during surgeries that expose various fat depots. Expanding the sample size of adipose biopsies would promote the establishment of a human adipose sample bank covering different age groups, genders, and metabolic states, which would improve relevant clinical metabolic and aging phenotype data to clarify the relationship between adipose aging and systemic metabolic homeostasis and aging, and help to find the most stable, representative, and clinically feasible adipose aging indicators and clinical intervention methods. Ultimately, the goal is to achieve early intervention in adipose tissue aging to promote healthy aging with the concept of “treating diseases before they occur.”

It should be noted that with the rise of high-throughput sequencing, the construction of cloud platforms, and the advancement of big data analysis, these technologies will accelerate the development of adipose aging assessment [184]. Genome-wide association studies, whole blood genomics, conventional transcriptomics, non-coding RNA, DNA methylation, proteomics, single-cell sequencing technologies, spatial transcriptomics, gut microbiota, and other research will gradually be applied to the field of adipose aging. Emerging sequencing techniques such as single-cell sequencing and spatial transcriptomics are likely to be incorporated into aging cohort studies in the future. More assessment parameters included means larger and more complexed data volumes generated, which may make adipose aging assessment expensive and difficult to promote. Differences in geographical locations of population cohort studies, parameters collected by various medical institutions, diverse data collection methods, and data analysis algorithms would lead to heterogeneity between databases, which may reduce research compatibility, increase scientific barriers, and limit the generalizability and scientific value of data from single centers. Thus, it would be crucial to establish unified adipose aging inclusion and assessment standards and standardize data management principles.

## Future work frame

### Key recommendations for adipose tissue aging biomarkers

Based on expert discussions, adipose tissue aging biomarkers are recommended from three dimensions: fat function, imaging, and body fluids, including functional changes reflecting fat content, distribution, thermogenic capacity, and organ lipid deposition, imaging changes in fat volume and composition, and changes in blood and urine components. These markers are suitable for clinical application and will be verified in different age cohorts in the future (Table 2). We have appended the abbreviations in this consensus and their full names in Table 3.

**Table 2.** Biomarker recommendations for adipose tissue aging

Dimension	Biomarker	Test method	COR	LOE
Functional markers	Body mass and composition	BMI, WC, BFP	IIa	A
	Fat function	Energy storage and expenditure, thermogenesis	IIb	B
	Fat redistribution	Fat accumulation and lipid deposition in multiple organs	IIb	B
Imaging markers	SAT, VAT, blood flow	US, DEXA, CT, MRI, CDFI	Ia	A
	Brown fat activity	IRT, ^18^F-FDG-PET/CT, CrCEST MRI	Ia	A
	Thickness and content of fat around organs	CT, MRI, MRE	IIb	B
	Content of fat deposition	B-ultrasound, CT, MRI, Fibroscan, MRI-PDFF	IIb	B
Humoral markers	Leptin, adiponectin, resistin	Plasma/ELISA	Ia	A
	Glutamine, creatine, ceramide, 5-HEPE	Plasma/mass spectrometry	IIb	B
	8-oxyguanosine, 8-oxodeoxyguanosine, PGE-M	Urine/ELISA, mass spectrometry	IIb	B

The corresponding color grades in Table 2 are consistent with Table 1.

**Table 3.** Abbreviations in this consensus

5-HEPE	5-hydroxyeicosapentae-noic acid
^18^F-FDG-PET/CT	[^18^F] Fluorodeoxyglucose positron emission tomography and computed tomography
AD	Alzheimer’s disease
BAT	Brown fat tissue
BCAA	Branched chain amino acid
BFP	Body fat percentage
BHB	β-hydroxybutyrate
BIA	Bioelectrical impedance analysis
BMAT	Bone marrow adipose tissue
BMI	Body mass index
CCTA	Coronary computed tomography angiogram
CD	Crohn’s disease
CF	Crawling fat
CKD	Chronic kidney disease
cMAT	Constituent marrow adipose tissue
CT	Computed tomography
CVAI	Chinese visceral adiposity index
DEXA	Dual energy X-ray absorption
EAT	Epicardial adipose tissue
ECM	Extracellular matrix
FDF-21	Fibroblast growth factor 21
GFR	Glomerular filtration rate
IL6	Interleukin-6
IMAT	Intermuscular adipose tissue
IRT	Infrared thermography
LDL	Low density lipoprotein
MAT	Mesenteric adipose tissue
MASLD	Metabolic dysfunction-associated steatotic liver disease
MASH	Metabolic dysfunction-associated steatohepatitis
MCFI	Mesenteric crawling fat Index
MHO	Metabolically healthy obesity
MRI	Magnetic resonance imaging
MUO	Metabolically unhealthy obesity
NEFA	Nonestesterified fatty acid
OPN	Osteopontin
PET-CT	Positron emission tomography/computed tomography
PFC	Pancreatic fat content
PFD	Pancreatic fat deposition
PRDM16	PR-domain containing 16
PTHrP	Parathyroid hormone-related protein
RBP-4	Retinol-binding protein 4
rMAT	Adjustable marrow adipose tissue
RVR	Renal vascular resistance
SAT	Subcutaneous adipose tissue
SLD	Steatotic liver disease
Srebp1c	Sterol regulatory element-binding protein 1
SUV	Standard uptake value
T2D	Diabetes mellitus type 2
TAMA	Total abdominal muscle area
TGF-β	Transforming growth factor-β
Treg	Regulatory T cells
VAT	Visceral adipose tissue
vBMD	Volumetric bone mineral density
VFA	Visceral fat area
VLDL	Very low-density lipoprotein

It has to be noted that aging and obesity share certain molecular markers, which reflect the pathophysiological intersection between aging and obesity, including common mechanisms such as insulin resistance and oxidative stress due to chronic low-grade inflammation and metabolic dysregulation. Meanwhile, there are distinct features between the two processes. For example, aging is characterized by visceral fat accumulation and subcutaneous fat atrophy, whereas obesity typically manifests as global adipose tissue hyperplasia. At the molecular level, aging is driven primarily by cellular senescence (e.g. senescence-associated secretory phenotype (SASP) factors, p16INK4a), while obesity is associated with adipocyte hypertrophy/hyperplasia (e.g. upregulated adipogenesis-related markers). Future work is warranted to analyze distinct molecular signatures, metabolic pathways, and physiological changes unique to aging and obesity to elucidate definitive biomarkers to reliably differentiate aging, adiposity, and fat distribution.

### Roadmap for adipose tissue aging biomarker research in China

Through expert consensus, these biomarkers will be further verified in cohorts in the future to promote high-quality adipose tissue aging marker research in humans. A translational link between basic and clinical research will be established to accelerate the research process of adipose tissue aging intervention and contribute to the healthy aging of adipose tissues in human.

The action framework for adipose tissue aging marker research in China includes:

(1) Promoting the standardization and expansion of cohort establishment for the “adipose tissue aging research plan of wide regions and large sample sizes” in China;
(2) Establishing an adipose tissue aging marker network suitable for the Chinese population to identify critical turning points of adipose tissue aging and clarify vital time points for intervention;
(3) Promoting the development of biomarker detection technologies to establish an adipose tissue biological age measurement system and disease prediction models based on artificial intelligence;
(4) Combining advancements from industry, education and research to promote the establishment and application of guidelines and standards for adipose tissue aging marker research in China.

## Key messages

The Aging Biomarker Consortium established a multidimensional biomarker framework for assessing adipose tissue aging, comprising functional, imaging-based, and humoral markers.

Functional biomarkers of adipose aging include WAT accumulation, abdominal and subcutaneous adipose tissue re-distribution, visceral adipose tissue dysfunction, blunted thermogenesis, and increased ectopic lipid deposition in vital organs that drives metabolic dysfunction.

Imaging-based biomarkers of adipose aging including increased VAT volume, decreased subcutaneous fat thickness, decreased BAT volume and activity, reduced blood flow in adipose tissue, increased ectopic lipid deposition, which can be measured with US, DEXA, MRI, CT, PET-CT, and infrared thermography.

Humoral biomarkers of adipose aging, including serum adipose-derived proteins or metabolites such as leptin, adiponectin, resistin, creatine, ceramides, and some urine markers related to aging and obesity, such as 8-oxo-guanine, 8-oxo-deoxyguanosine, 8-isoprostane, and PGE-M.

Research on evaluating the biological age of adipose tissue is still in its infancy and molecular and pathological detection of adipose tissue biopsies may be the best method that can provide high-throughput sequencing resources and accurate adipose aging information.

Future work should focus on identifying the most stable, representative, clinically feasible adipose aging biomarkers, as well as developing strategies to achieve early intervention in adipose tissue aging and promote healthy aging.
